# Applying the “SOTEC” framework of sociotechnical risk analysis to the development of an autonomous robot swarm for a public cloakroom

**DOI:** 10.1111/risa.17632

**Published:** 2024-08-23

**Authors:** Peter Winter, John Downer, James Wilson, Dhaminda B. Abeywickrama, Suet Lee, Sabine Hauert, Shane Windsor

**Affiliations:** ^1^ School of Sociology, Politics and International Studies (SPAIS) University of Bristol Bristol UK; ^2^ Dyson Institute of Engineering & Technology Malmesbury UK; ^3^ Department of Computer Science The University of Manchester Manchester UK; ^4^ School of Engineering Mathematics and Technology University of Bristol Bristol UK; ^5^ School of Civil, Aerospace and Design Engineering Bristol UK

**Keywords:** autonomous robotic swarms, safety assurance, scenario analysis, sociotechnical risk analysis, SOTEC

## Abstract

The past decade has seen efforts to develop new forms of autonomous systems with varying applications in different domains, from underwater search and rescue to clinical diagnosis. All of these applications require risk analyses, but such analyses often focus on technical sources of risk without acknowledging its wider systemic and organizational dimensions. In this article, we illustrate this deficit and a way of redressing it by offering a more systematic analysis of the sociotechnical sources of risk in an autonomous system. To this end, the article explores the development, deployment, and operation of an autonomous robot swarm for use in a public cloakroom in light of Macrae's structural, organizational, technological, epistemic, and cultural framework of sociotechnical risk. We argue that this framework provides a useful tool for capturing the complex “nontechnical” dimensions of risk in this domain that might otherwise be overlooked in the more conventional risk analyses that inform regulation and policymaking.

## 1 INTRODUCTION

### 1.1 Autonomous robotic swarms and safety assurance

Autonomous robotic swarms are multi‐agent robot systems that exhibit emergent behavior through environmental and agent‐agent interaction not explicitly engineered in the system (Winfield et al., 2005). As these systems have advanced, they have garnered growing interest for various scientific, industrial, commercial, and military applications (Schranz et al., 2020). Illustrative of this growing interest, and the focus of this article, is a robot swarm being developed by a team of engineering researchers at the University of Bristol (UoB), United Kingdom, for the purpose of transporting personal belongings in a public cloakroom (Abeywickrama et al., 2023; Jones et al., 2020, 2022).

**FIGURE 1:**
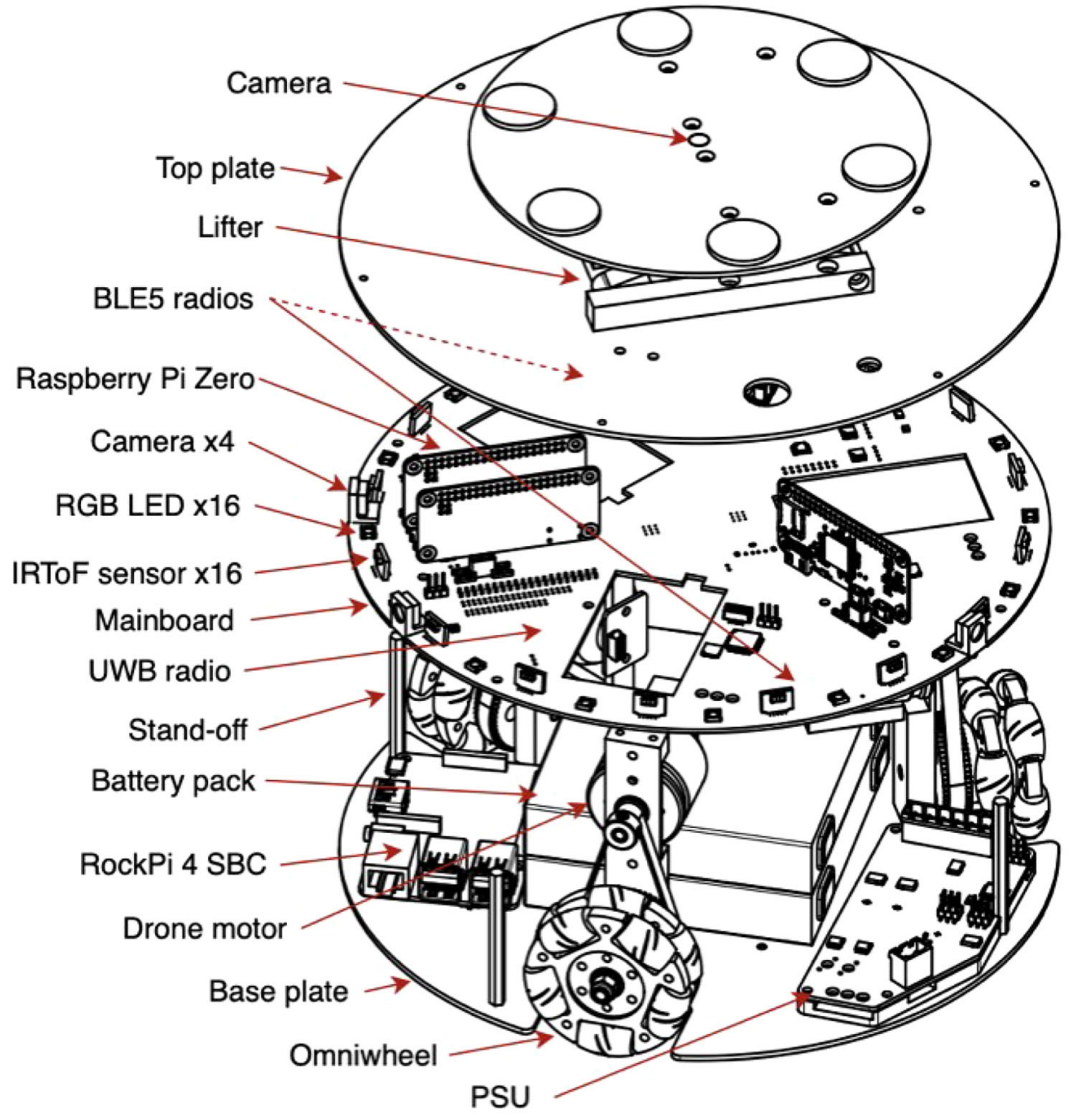
Exploded view of a swarm robot chassis showing major components (Jones et al., 2022: 14).

As of writing, the UoB team's experimental swarm consists of 20 custom‐built robots. Each is about 10 by 10 inches in size and is equipped with a variety of limited range sensing and communication capabilities, including cameras, distance sensors, and Bluetooth chips (Figure 1). Using a combination of procedural rules and artificially evolved behavior trees, the swarm engineers have programed each robot to react to local interactions with other robots and their environment, coordinating their actions in ways inspired by swarm behaviors in nature (Jones et al., 2020). The goal is to design an intelligent swarm of robots that can efficiently receive belongings from a human attendant, deposit them in a controlled storage area, and retrieve them when required. Robots in the swarm must be capable of navigating enclosed small spaces (such as narrow corridors) and miscellaneous obstacles (such as dropped boxes and broken‐down robots), all while avoiding collisions and bottlenecks (Jones et al., 2020).

To this end, each robot is equipped with multiple sensors. The first is its “vision system,” which comprises four cameras for a 360° horizontal view and a fifth camera that looks vertically upwards. The second consists of proximity sensors, which surround the perimeter of each robot, allowing the robots to measure distance and detect the state of their environment. The third sensor system is a suite of discrete instruments that collects environmental information, such as temperature, pressure, and humidity, as well as information on the state of the robot's internal systems, such as its power supply and wheel position. The fourth system is for communication. It consists of WiFI for software updates and Bluetooth components that allow the robots to return belongings to attendees.

This design goal may sound reasonably straightforward, but it presents complex assurance challenges. For example, existing standards and best practices for the design of autonomous systems in the industrial robotics domain (e.g., ISO/TS, 2016, 2006/2) and the service robotics domain (e.g., ISO, 13482) may not be useful or relevant to distributed and evolving swarms. Distributed systems are problematic because these standards tend to be oriented to construing safety and performance as a property of individual robots as opposed to the system more broadly (Abeywickrama et al., 2023, 2024). This gives rise to complex difficulties. For example, research into the nascent field of human‐swarm interaction (HSI) often argues that a single human is unable to monitor or control large groups of autonomous robots, thus calling attention to the fact that monitoring or controlling work is required from a team of human operator experts (Kolling et al., 2016; Brown et al., 2016; Carrillo‐Zapata et al., 2020; Divband‐Soorati, 2021; Wilson & Hauert, 2022). Evolving systems, meanwhile, are problematic because standards tend to mandate predictable and fixed behaviors rather than emergent and changing behaviors (Abeywickrama et al., 2023, 2024; Farnell et al., 2019).

It is not surprising, therefore, that using an autonomous robotic swarm in a public cloakroom is likely to create unique risks and corresponding assurance challenges (Soltanzadeh, 2022). So it was, for example, that one risk analysis of the system (Abeywickrama et al., 2023) identified the possibility of individual robots accumulating, and thereby blocking, critical areas such as fire exits (see also Milner et al., 2022: 270; Carrillo‐Zapata et al., 2020: 10; Hunt & Hauert, 2020: 421).

Indeed, studies are articulating an ever‐growing range of distinctive risks that arise from deploying autonomous robotic swarms with multi‐agent emergent behavior. For instance, the possibility that degradation or faults rapidly cascade through the system leading to compounded failures (Abeywickrama et al., 2023). It is, therefore, imperative that the risks, uncertainties, and novelties of such swarms be analyzed and managed effectively.

### 1.2 Existing risk analysis approaches to autonomous robot swarms

Studies of the management of risk in autonomous systems span a wide range of applications and domains, such as automotive, medicine, and military (e.g., Brundage et al., 2020; Hawkins et al., 2021; Neto et al., 2022). Much of this work is aimed at articulating risks to facilitate their identification and mitigation. In this scenario, risk analysis typically follows the guidelines of traditional safety assurance methods. This involves pinpointing potential hazards—meaning any foreseeable dangers—assessing how likely these hazards are to happen and determining ways to prevent or lessen their impact (ISO 12100, 2010). From this perspective, a hazard represents a potential source of harm, whereas risk encompasses the probability of that harm occurring and its potential consequence (ISO 12100, 2010; ISO, 2018; Purdy, 2010).

Hunt and Hauert (2020) established a foundation for analyzing the risks of robot swarms by crafting a “safe swarm checklist” intended to help engineers identify hazards early in the design process. The checklist outlined 10 questions to be considered before any swarm was deployed in the real world. Questions 1 and 2 pertained to ethical and legal concerns; questions 3 and 4 related to accountability and user interaction; questions 5–8 focused on physical hazards the system might pose, and questions 9 and 10 related to swarm security in terms of being hacked and subverted by malicious actors.

Abeywickrama et al. (2023) have proposed a different approach to assessing the risks of autonomous swarms via an assurance process they call “AERoS” (“Assurance of Emergent behavior in autonomous Robotic Swarms”), which is designed to analyze system‐level safety requirements. Applied in synthesis with expert judgment of system safety requirements, AERoS is intended to enable the identification of system‐level risks in respect to four categories of safety requirement: “performance,” “adaptability,” “human safety,” and “environment.”

Other studies of risk in autonomous swarms specifically focus on the components that implement security features, such as access controls and level of security assurance. Hernández‐Herrera et al. (2018), for example, applied “failure mode and effect analysis” (FMEA) to identify possible cybersecurity vulnerabilities in swarm robots. Their study focuses on risks relating to a certain type of cyberattack, but it demonstrates how FMEA can be applied to enhance engineers’ understanding of design‐related failure modes (Hernández‐Herrera et al., 2018: 323).

Research in this domain has been devoted to the processes of fault detection (Akhter et al., 2022; Lee et al., 2022; O'Keeffe et al., 2018; Qin et al., 2014; Tarapore et al., 2020; Winfield & Nembrini, 2006). Such studies tend to focus on the development of fault‐aware swarm systems, but risk is often implicitly or explicitly present in their analyses. Akhter et al. (2022), for example, performed a “hazard and risk assessment” to help them identify and classify potential hazards and “failure modes” in their swarms, so as to better define “resilience” goals. Their method is based on the classic risk assessment model, which involves the evaluation of two key factors: probability and consequences (ISO, 2018). This involves assessing risks based on the probability of an event occurring and the potential consequences or impact of that event (commonly known as the “probability vs. consequences” or “likelihood vs. severity” model) (ISO, 2018; Purdy, 2010), allowing them to assess different risks that each hazard poses based on the likelihood of severity, exposure, and recoverability.

### 1.3 Shortfalls

Although the focus on human factors engineering (e.g., Hunt & Hauert, 2020) and HSI design (e.g., Carrillo‐Zapata et al., 2020) in these studies represents a significant development in the risk analyses of autonomous robot swarms, improvements are still needed. Below, we outline four neglected requirements for these systems to be safe and effective.

1. Existing approaches to assessing risks in swarms tend to view failure as a chain of events that links a failure in an individual robot or a robot in a group of robots (the “neighborhood”) with some type of hazard (Abeywickrama et al., 2023; Akhter et al., 2022; Hernández‐Herrera et al., 2018). This view is premised on the understanding that an accident can be traced back in time to a primary failure (such as the blocking of critical areas such as a fire exit) and is perhaps best exemplified by the dominance of FMEA. FMEA, however, has garnered some criticism (Congenius, 2021; Zhang & Liu, 2023). Zhang and Liu (2023), for instance, argued that it ignores in‐use risks of the technology by consumers and does not consider risks arising from certain types of interdependencies and non‐linear interactions between different levels of complex systems, human factors, and the environment.
2. Existing research predominantly focuses on technical risk factors and neglects other dimensions of risk, such as human, organizational, social, political, ethical, and environmental factors. The latter are important, however, especially human factors, which play an increasingly important role in the success or failure of swarm systems (Abeywickrama et al., 2024).
3. Much of this research tends to be either theoretical or simulation based (e.g., Abeywickrama et al., 2023; Lee et al., 2022). As such, it fails to capture many conditions of HSIs in the real world.
4. Existing research closely construes “risk” quantitatively, implying that both its probability and its consequences can be accurately measured as some form of probabilistic technique, such as probabilistic risk assessment (PRA) (Farnell et al., 2019). Yet this construal is incommensurate with the organizational complexity and unpredictable environments in which autonomous systems are deployed (Macrae, 2022). Construing risk more expansively invites a shift away from thinking about risk primarily as a matter of probability in scenario planning and instead takes risk as a matter of plausibility, illuminating how analyses can privilege both its quantitative and its qualitative dimensions (Abeywickrama et al., 2024; Glette‐Iversen et al., 2022; Perrow, 1999; Purdy, 2010; Ramírez & Selin, 2014). This model of plausibility values the importance of stories and qualitative information, which considers the plurality of views, contextual conditions, and experiential evidence instead of relying on quantitative approaches alone (captured in formulae, statistics, regressions, etc.) (Ramírez & Selin, 2014). Thus, based on plausibility rather than probability in scenario planning and helped by Glette‐Iversen et al.’s (2022: 1) analysis of the term in the literature, we interpret the concept of “plausibility” in its risk analysis context as a tool for measuring the uncertainty of an event—one that combines the likelihood of an event occurring with judgments about the quality or strength of the supporting knowledge.

Many of these shortfalls are not unique to the autonomous swarm domain and largely reflect the broader landscape of risk analysis of autonomous systems in general. To better account for these neglected dimensions of risk in autonomous systems—especially as they pertain to complex human, social, and organizational relationships—scholars have increasingly endeavored to capture its “sociotechnical” aspects. For example, research by Elish (2019), Salmon et al. (2020), and Macrae (2022), Stilgoe (2018), Winfield and Jirotka (2017) advocate for the development of a “human factors” approach to assessing autonomous systems.^1^ Such approaches were bolstered in 2018 by a high‐profile accident with an Uber autonomous vehicle that struck and killed a pedestrian crossing a road (NTSB, 2019). The collision was attributed to operator error, but investigations revealed important underlying organizational and systemic factors (Macrae, 2022). The accident illustrated something long‐understood in many technological domains: that the causes of failures are rarely reducible to technical criteria alone. These systems are “…designed, developed, built, deployed, maintained, supervised, operated, and governed by people,” as Macrae (2022: 2001) puts it, “and that those people operate within complex social, cultural, and organizational processes” (see also: Pettersen Gould, 2021; Reason, 1997).

This more “holistic” and human centered approach to safety has resulted in the recent development of various sociotechnical approaches to the risk analysis of autonomous systems (Loh et al., 2020; Macrae, 2022; Sethu et al., 2022) as well as autonomous robot swarm projects with associated sociotechnical considerations (Hunt & Hauert, 2020; Kaasinen et al., 2022; Khopkar, 2020; Weber, 2021).

Recent research by this team calls for a hybrid approach, combining technical research and sociotechnical research (Abeywickrama et al., 2024). The focus of technical research is on the identification of technological risks and the application of engineering principles to solve these risks, such as assigning probabilities to scenarios for risk management. This approach has long been integral to scenario planning in risk research and is central to our AERoS process. The AERoS process involves analyzing swarm behavior using a probabilistic finite state machine: probabilities are applied to evaluate different scenarios and verify that the emergent beaviors of the swarm meet safety requirements through six assurance activities (Abeywickrama et al., 2023). This process builds on the work of Hawkins et al. (2021) on integrating assurance activities into the development of autonomous systems (“AMLAS”). This specification provides a detailed template for technical research. However, the intent is that both the technical and sociotechnical nature of hazards and risks are required if we are to build a better understanding of risk. For this reason, this article can be seen as a companion to AERoS, moving from probability in scenario planning toward a deeper dynamic of plausibility in scenario planning: one that includes organizational, social, psychological, political, and cultural aspects to help render scenario work more robust (Ramírez & Selin, 2014).

**FIGURE 2:**
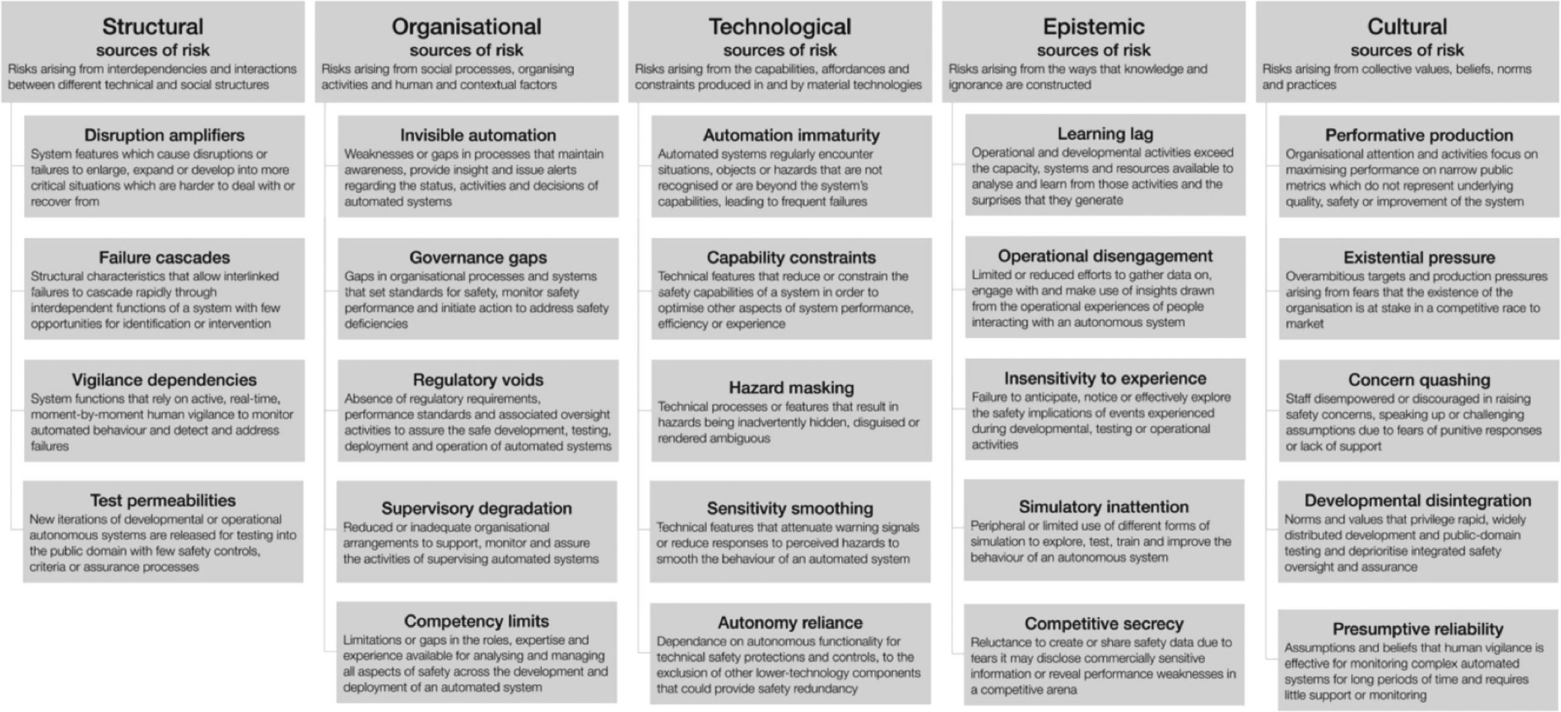
SOTEC: structural, organizational, technological, epistemic, and cultural sources of sociotechnical risk and patterns of failure in autonomous and intelligent systems (from Macrae, 2022: 16).

In keeping with this research agenda, the following argument invokes Macrae's (2022) structural, organizational, technological, epistemic, and cultural (“SOTEC”) framework of sociotechnical risk to identify and analyze five sources of risk in an autonomous robot swarm operating a public cloakroom (Figure 2). According to Macrae (2022), safety teams and risk researchers need to draw on models, methods, and knowledge beyond technological risks and their underlying functionality to encompass human and contextual factors (i.e., “SOTEC”). Although AERoS (Abeywickrama et al., 2023) has provided us with the technological aspects of autonomous robot swarm safety, we need to contextualize it with these other sources of risks. Moreover, through these sources of risk, regulatory requirements can be recommended, with the eventual goal of achieving both better design and risk management processes.

### 1.4 Aims and research questions

**FIGURE 3:**
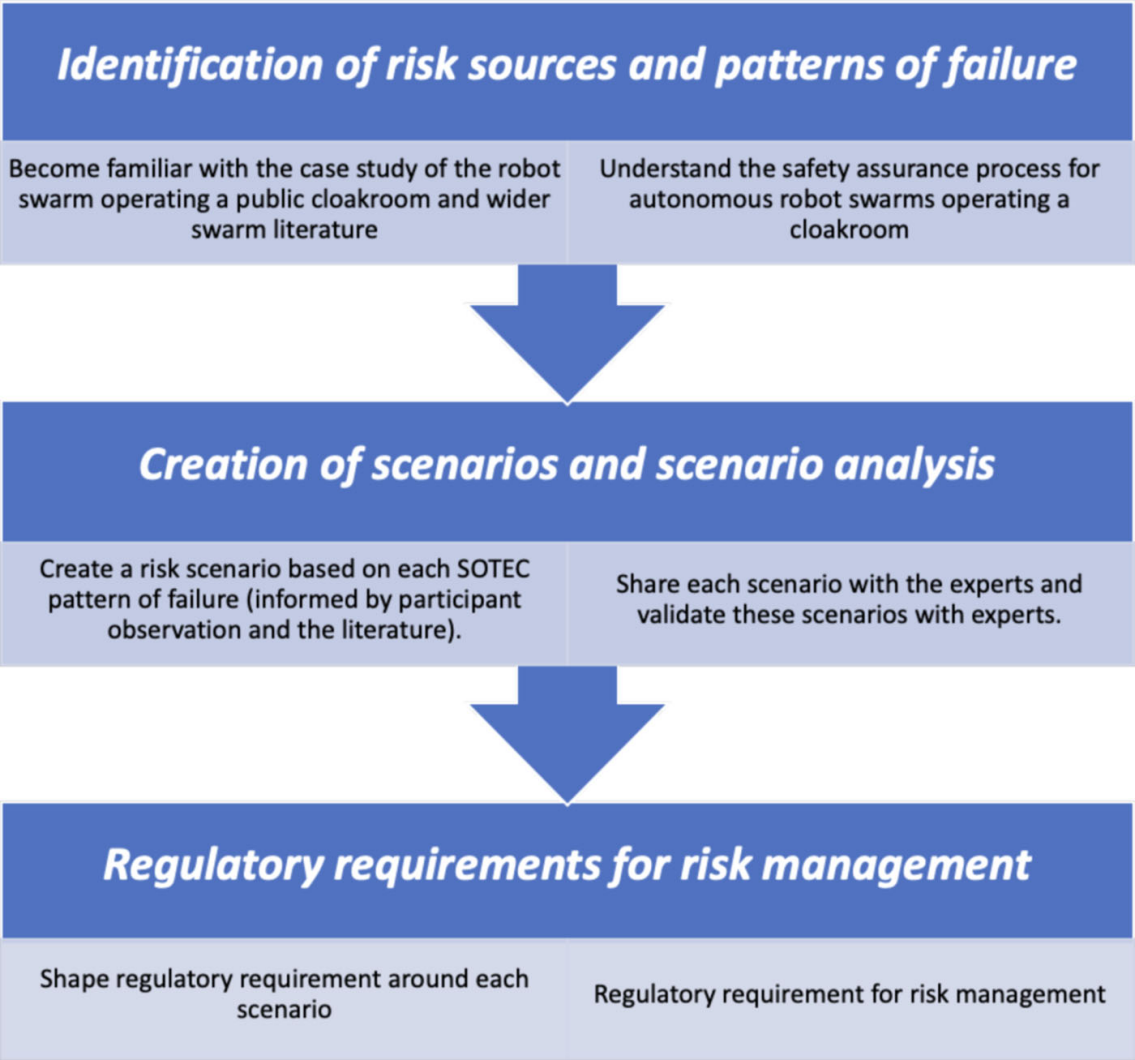
Application of structural, organizational, technological, epistemic, and cultural (SOTEC) for risk analysis of autonomous robot swarms.

This article has two aims. The first is to identify key sources of sociotechnical risk and their patterns of failure in a case study centered on the operations of a robot swarm in a public cloakroom. The second is to use these sources of risk to inform regulatory requirements for such operations. In this article, therefore, we address two research questions: (i) What sources of sociotechnical risk and patterns of failure can emerge in the design, development, and deployment of autonomous robot swarms? and (ii) What regulatory requirements are applicable? We also outline a qualitative research method to answer these research questions (Figure 3).

## 2 METHODOLOGY

### 2.1 The SOTEC framework

Macrae (2022) established the SOTEC framework on the back of a detailed study of the fatal 2018 Uber self‐driving crash for analysis of sociotechnical sources of risk in autonomous and intelligent systems. He demonstrates the application of the framework to facilitate the key building blocks of a learning infrastructure that supports the governance of safety and the management of risk across the autonomous systems lifecycle (spanning from design to development to deployment to operation).

The fundamentals of SOTEC lie in the field of disaster studies, which is concerned with exploring how technological complexity combines with human entanglements to create conditions for the emergence of novel, unexpected, and surprising behaviors (Downer, 2023; Perrow, 1999; Weick & Sutcliffe, 2001). The framework's main elements are a diverse range of interrelated sources of risk (“SOTEC”), expressed and consolidated through patterns of failure (Figure 2). According to Macrae (2022), each core source of sociotechnical risk is illustrated by a range of particular patterns of failure that make up and consolidate the source. For example, “structural” sources of risk in autonomous and intelligent systems are represented by four indicative patterns of sociotechnical failure, specifically: “disruption amplifiers,” “failure cascades,” “vigilance dependencies,” and “test permeabilities” (Macrae, 2022). Similarly, “organizational” sources of risk are represented by five patterns of sociotechnical failure, specifically: “invisible automation,” “governance gaps,” “regulatory voids,” “supervisory degradation,” and “competency limits” (see Figure 2 for a full list of the sources of risk and patterns of failure).

SOTEC is a systematic qualitative research approach to risk assessment. It aims to fill a void in traditional approaches to qualitative studies in risk analysis with its instructional sources and patterns of sociotechnical risk, where most applications of qualitative research remain “unsystematic” (Pettersen Gould, 2021: 462). By helping identify sociotechnical sources of risk and patterns of failure, moreover, the framework also provides a tool for framing context‐specific regulatory requirements. Hence, our application of SOTEC here is twofold. We use it to identify sources of risk and patterns of failure (such as “disruption amplifiers” and “concern quashing”) to swarm robots in the cloakroom, and we use it to inform relevant regulatory requirements for regulatory decision making.

This is the first time that a sociotechnical risk analysis framework has been proposed for analyzing sociotechnical risk of autonomous robot swarms. In order to support our approach, our application of the framework is also supplemented in the article by a process that describes the necessary steps required to: (1) identify risk sources and risk patterns; (2) building risk scenarios; and (3) write regulatory requirements, which is also novel and aimed at producing transparency and replicability. As Macrae (2022) does not offer a procedure or process to show how the framework should be applied, we provide below a brief description of our process, which follows a three‐stage iterative framework, comprising the identification of risk sources and risk patterns, risk scenario building, and risk‐informed regulatory requirements (Figure 3).

#### 2.1.1 Identification of risk sources and patterns of failure

This stage consisted of two tasks. The first was to become familiar with the case study—the robot swarm operating a public cloakroom—so to understand its safety assurance requirements. This involved the first author's (PW) ethnographic participant observation of three collaborating researchers: a computer scientist with expertise in producing requirements specifications for AI systems (DA) and two swarm engineers with expertise in developing swarm systems (JW; SL). The researchers were developing a process of safety assurance via Microsoft Teams online. This collaboration resulted in the AERoS safety assurance process (Abeywickrama et al., 2023). The second task was then to work with this group and introduce them to SOTEC to identify the sociotechnical risks that could potentially cause or culminate in harm posed by the swarm in the cloakroom. To help ensure a comprehensive list, five sources of risk from the SOTEC framework were introduced by the first author (PW) to the research teams. This process involved a verbal description of a SOTEC “risk dimension” and its definition to the researchers (so, for example, the first author raised the matter of *structural sources of risk*, followed by the definition: *risks arising from interdependencies and interactions between different technical and social structures* [Macrae, 2022: 7]). Each definition was then illustrated with context using the Uber 2018 crash as an example (such that, for instance, for “structural sources of risk,” PW explained that the structure of a sociotechnical system is made up of many interacting elements and that a breakdown in the interactions between these elements across different parts of the system contributed to the Uber crash). This process was then repeated until each source of sociotechnical risk was covered. At this point, PW mapped each of the five sources onto the swarm case study.

Once a sufficient understanding of sociotechnical risks and failures was gained between swarm engineers, PW moved onto the second stage: risk scenario creation.

#### 2.1.2 Creation of scenarios and scenario analysis

The next stage aimed to produce a set of risk scenarios for an autonomous robot swarm in a public cloakroom, based on each pattern of sociotechnical risk from the SOTEC framework. This was carried out through a process of “scenario analysis.”

Scenario analysis—through which engineers consider a spectrum of possible futures to help manage uncertainty—has a long history in risk research, where it has been closely associated with requirements engineering (Brown et al., 2000; Hausfather & Peters, 2020; Maia, 2013; Wright et al., 2019). It works by engaging relevant stakeholders in a structured approach that helps them identify problem situations that could significantly influence the future application of the technology. The stakeholders are then asked to reflect on these problems to create a diverse range of plausible future scenarios. From here they are asked to analyze the potential impacts and implications of each scenario for different parties, including developers, end users, affected communities, the environment, and society as a whole. The process encourages stakeholders to identify novel risks, challenges, and uncertainties associated with each scenario and also to develop appropriate response strategies (Wright et al., 2019).

Scenario analysis has many advantages. It helps experts distance themselves from present arrangements enabling a space for criticism. It facilitates future‐orientated action based on information about different scenarios. And it informs policy development, allowing policymakers to make more informed decisions and develop policies that are better equipped to address complex and uncertain challenges (Brown et al., 2000; Eden et al., 2013; Selin, 2011; Wright et al., 2019). Most significantly for our purposes, it is easily aligned with the SOTEC framework because it allows for a combination of technical and social factors to be considered, accompanied by their risks and uncertainties.^2^

As with any method, scenario analysis has its limitations. It is susceptible to participant assumptions and biases (Schwartz, 1996). Being qualitative, it makes it challenging to compare or prioritize scenarios objectively (Van Der Heijden, 2005). It lacks formal mechanisms for quantifying probability, which complicates the prioritization of risks (Bishop & Hines, 2017). It has limited predictive accuracy due to its emphasis on exploring possible futures (Wright et al., 2009). And it is unable to capture important interactions (business, political, and financial) that may influence scenario practice (Ramírez & Selin, 2014). This latter limitation, in particular, raises fundamental questions about the limits of knowledge, the constraints of knowledge, and the power of imagination (Ramírez & Selin, 2014).

Importantly for this article, the case study of the cloakroom presented in the prior AERoS publication (see Abeywickrama et al., 2023), which helped contextualize and inform our scenario analysis, had already led the participants to imagine a range of relevant risks and uncertainties and linking them to patterns of failure. This provided a base on which the SOTEC analysis was able to build.

Research by Stilgoe et al. (2013) shows a renewed interest in scenario analysis as a foresight tool, especially for researchers looking to generate plausible outcomes and plausible futures for anticipatory governance (considered to be a cornerstone of responsible innovation) (Nordmann, 2014; Winter & Carusi, 2022). As such, our approach resonates with work by Maia (2013) and Stilgoe et al. (2013), which construe scenarios as stories about possible futures, emphasizing what *could* happen rather than what *will* or *should* happen as a rational, systematic approach to managing an uncertain future.

The scenarios developed as part of this study involved a two‐step process. First, using SOTEC as a guide, PW built scenarios around each pattern of failure (a process that was informed by participant observation and literature analysis). PW then presented each scenario to the experts to gauge their opinion on it and its plausibility. The scenarios were then adjusted in light of their feedback.

#### 2.1.3 Regulatory requirements for risk management

The third stage of our analysis involved the formulation of regulatory requirements reflecting each identified pattern of failure. Such that, for example, a regulatory requirement for managing the risk of “disruption amplifiers” might be “the identification and prevention of disruption amplifiers.” The eventual aim was to create regulatory requirements tied to specific patterns of failure. Many of these were what Nyvik et al. (2021) refer to as “soft” requirements, identifying goals to be achieved rather than specific mechanisms of achievement. For example, in the case of “disruption amplifiers” (i.e., “system features which cause disruptions or failures to enlarge, expand, or develop into more critical situations” [Macrae, 2022: 2006]), we have written the following “soft” regulatory requirement, identifying what goals are to be achieved in order to prevent or manage potential disruption amplifiers:

The system shall be able to identify anomalies in individual robots and prevent them from causing broader system disruptions. For instance, if a robot has a faulty radial camera that prevents it from detecting a missing container, a potential strategy could involve another robot with a functioning camera identifying the fault. The functioning robot would then instruct the faulty robot that it is no longer carrying a container. This communication would enable the faulty robot to switch to a safe state, move to a secure location, and send warning signals to the swarm and human operators via hazard messages or activated warning lights.

(See Table 1, Structural Sources of Sociotechnical Risk on Page 27).

**TABLE 1:** Structural sources of sociotechnical risk.

*Risks arising from interdependencies and interactions between different technical and social structures*		
Patterns of failure	Scenario example	Regulatory requirements
**Disruption amplifiers**		**Identification and prevention of disruption amplifiers**
“System features which cause disruptions or failures to enlarge, expand or develop into more critical situations which are harder to deal with or recover from” (Macrae, 2022: 8) Disruption amplifiers may also be thought of as a type of “interactive complexity,” where components may interact in unanticipated ways, perhaps because of failures or just because no designer anticipated the interactions that could occur (Perrow, 1999)	A disruption or failure in one part of the swarm system (such as a fault in the upward facing camera) prevents one individual swarm robot responding to a perceived hazard, impacting the performance of other swarm robots in ways that are hard to predict For instance, a robot with a failed camera acts as if it is carrying a container that is not there, taking precedence over other robots and cluttering the operating environment	The system shall be able to identify anomalies in individual robots and prevent them from causing broader system disruptions. For instance, if a robot has a faulty radial camera that prevents it from detecting a missing container, a potential strategy could involve another robot with a functioning camera identifying the fault. The functioning robot would then instruct the faulty robot that it is no longer carrying a container. This communication would enable the faulty robot to switch to a safe state, move to a secure location, and send warning signals to the swarm and human operators via hazard messages or activated warning lights.
**Failure cascades**		**Identification and prevention of failure cascades**
“Structural characteristics that allow interlinked failures to cascade rapidly through interdependent functions of a system with few opportunities for identification or intervention” (Macrae, 2022: 8)	A fault in one agent can cause other agents to fail	The system shall be able to monitor swarm‐level interactions, identify structural characteristics that cause interlinked failures to cascade, and intervene to prevent failures from propagating
Failure cascades may also be thought of as a type of “tight coupling,” wherein a failure cannot be isolated but brings about other failures that cascade through the system (Perrow, 1999)	For instance, a communication failure in one robot leads other robots to misconstrue its position, resulting in collisions and jams	
**Vigilance dependencies**		**State of vigilance**
“System functions that rely on active, real‐time, moment‐by‐moment human vigilance to monitor automated behavior and detect and address failures” (Macrae, 2022: 8)	An individual swarm operator does not remain attentive to the monitoring of faulty swarm behavior, and, as a consequence, is unable to intervene in time when the system fails	System safety shall not be predicated on unrealistic real‐time monitoring and vigilance by individual human operators
**Test permeabilities** “New iterations of developmental or operational autonomous systems are released for testing into the public domain with few safety controls, criteria, or assurance processes” (Macrae, 2022: 8)	Weaknesses in real‐world behavior arise in service due to insufficient testing or unrepresentative requirements	**Creation of processes for requesting and conducting testing** The system shall be subject to adequate pre‐deployment testing and assessment processes

Operationalized in the manner outlined here, the SOTEC framework offers a tool by which regulators might more optimally target their gaze and prioritize different risks (Black, 2005), and the requirements we identify should be understood as such. Their primary value lies in shaping regulatory thought in ways that promise greater context‐awareness (Nyvik et al., 2021: 1744).

In this way, our approach responds to a call by Winfield and Jirotka (2017) for a tool that better connects risks to regulation and, when accidents happen, facilitates more robust investigation. By construing regulatory requirements as open, dynamically evolving systems in and of themselves, we hope to encourage further research in a similar vein. We use SOTEC as a conceptual paradigm to identify risk scenarios and create corresponding regulatory requirements needed to mitigate these risks. Leveraged in this way, it operates as an instrument to challenge technical routines and organizational understandings, practices, and cultures (Huber & Rothstein, 2013; Macrae, 2014, 2022; Perrow, 1999).

## 3 APPLICATION EXAMPLE

### 3.1 Case study overview: the emergent behavior of an autonomous robotic swarm in a public cloakroom

To demonstrate the application of the SOTEC approach, it was applied to an imagined case study of an autonomous robotic swarm operating in a public cloakroom. The envisaged operator of this swarm is an event company, organizing bespoke events with 50–1000 attendees, which aims to deploy the robots to assist human attendees by depositing, storing, and retrieving their belongings (such as coats and scarves).

Although this application is imagined, the autonomous robot swarm was a tangible technology made with circuitry, metal plates, printed plastic, and screws—among many other things—being developed by the “swarm robotics group” at the Bristol Robotics Laboratory (BRL). The group included various roboticists (a professor, together with PhD students, technicians, postdoctoral researchers) exploring swarm behaviors that emerge from interactions between agents and their environment. The swarm they were developing was intended as part of a storage organization system with capabilities to collect, sort, and transport boxed contents in a cloakroom (Abeywickrama et al., 2023).

The requirements to which the swarm was being developed included: (i) That its robots should be able to transport containers weighing less than 2 kg (a *performance requirement*). (ii) That fewer than 10% of its robots would be stationary outside the delivery site at a given time (an *adaptability requirement*). (iii) That it be able to traverse environments with floor incline between 0° and 20° (an *environmental requirement*). (iv) That trained human operators be able to monitor 5–20 robots at a given time (*human safety requirement*).

**FIGURE 4:**
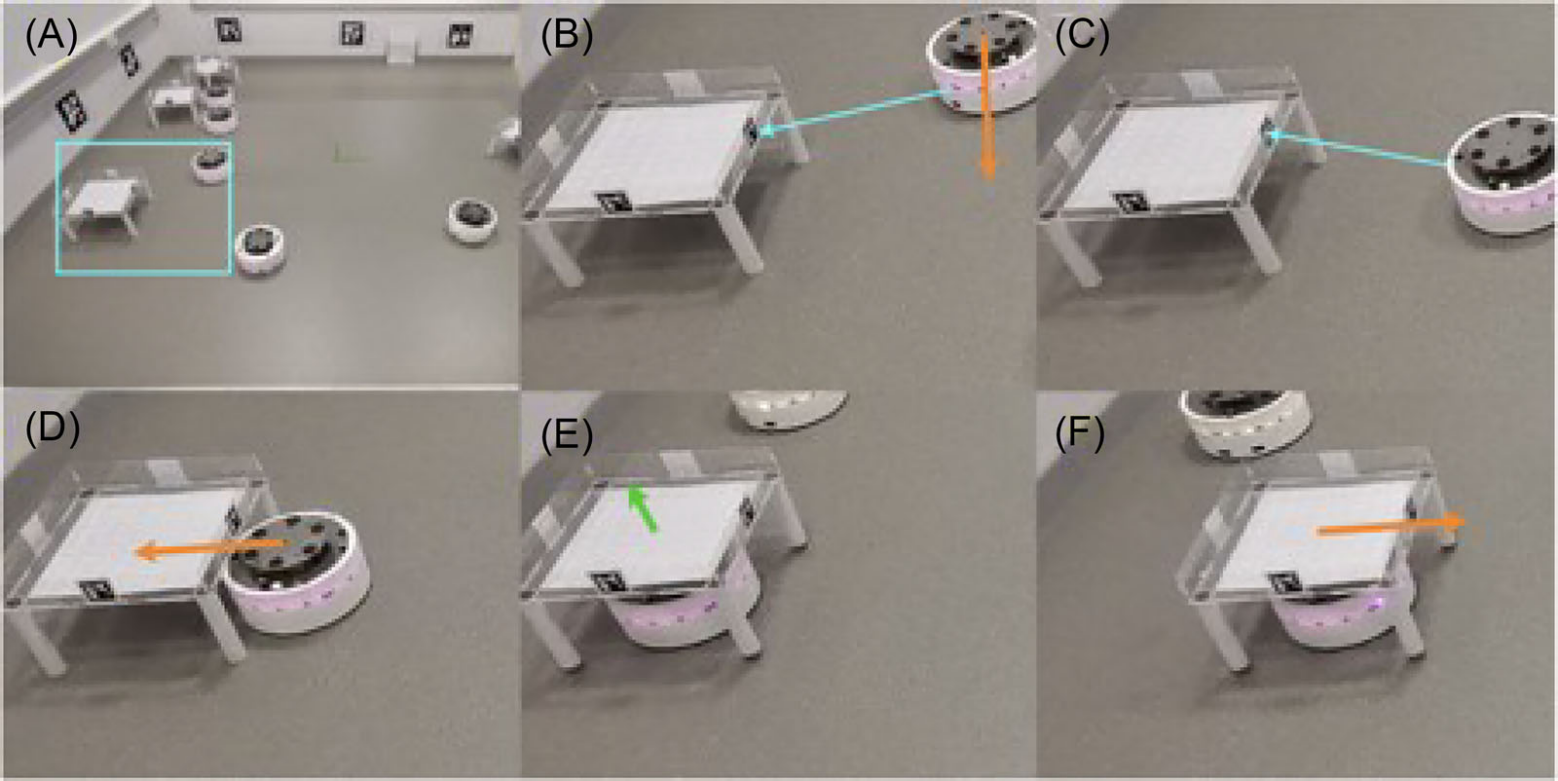
**The “DOTS” testbed**: Sequence showing a swarm robot picking up a carrier. (A) Overall view. (B) While exploring, a robot detects a carrier fiducial marker (blue arrow) and starts the pick‐up behavior. It moves to the pre‐dock position (orange arrow). (C) The robot is now facing the carrier and is in the optimal position for accurate pose estimation of the fiducial marker so (D) moves under the carrier and (E) centers itself using upwards‐facing camera and raises the lifting platform. Finally (F) the robot moves toward the drop zone. (Jones et al., 2022: 14).

The swarm was still very early in development at the time of writing, with no real‐world deployment and several challenges still to be overcome before it would be deployable. At this point in the development process, the engineers were using off‐the‐shelf simulation software (Gazebo 3D) to test the emergent behavior of the system in a 4 m x 4 m environment. This was an exact replica of the “DOTS” hardware testbed (Figure 4): A laboratory setting that the swarm robotics group at BRL had built and equipped with private 5G, motion capture, and multiple cameras to test a broad range of swarm applications (Jones et al., 2022).

At this early stage of the swarm's development, it was difficult to imagine precisely how it would be integrated into a real‐world cloakroom environment. Historically, the process of integrating novel technologies into the workplace has presented a variety of challenges (Bijker et al., 2012). These include organizational resistance to change (Capuccio et al., 2023); skill gaps and training needs (Reason, 1997); compatibility issues (Karahanna et al., 2006); cost concerns (Melville et al., 2004); security and privacy risks (Jonathan et al., 2020); cultural barriers (Beekhuyzen et al., 2005); and lack of leadership and support (Kumar Basu, 2015). As these challenges illustrate, such integration is more than simply a technical process but involves complex human, social, and organizational practices (Downer, 2023; Macrae, 2022). It was important, therefore, that the engineers anticipate these challenges in developing their cloakroom swarm.

The swarm's three main tasks—collection, storage, and retrieval—corresponded with three main areas: a collection area, where robots received belongings placed into empty containers (see Figure 1); a storage area, where the containers could be deposited; and a retrieval area, where the containers could be returned to the attendee's location. The robots would use the sensors described above to coordinate their tasks and identify the attendees (Figure 1), especially the Bluetooth sensor, which is essential for robots to: (1) identify the unique Bluetooth signal on the smartphone for each returning attendee and (2) deliver their belongings (complete and intact) to the attendee. For such tasks, each robot has been programed to keep within 2 m distance of a human (Abeywickrama et al., 2023).

These tasks raised numerous risk questions. For example, regarding the distance the robots should keep from attendees at the collection point. The robots were set to come within 2 m, but there was some uncertainty as to whether this was close enough for attendees to safely deposit their belongings, especially those with visual impairments or with disability issues. There were further questions regarding limitations on the size and weight of the belongings being deposited and about the number of belongings each container could contain. The state of the belongings created more uncertainties, especially in regard to wet items dripping water. In terms of the storage area, the engineers were unsure how much space the swarm would need to operate safely and efficiently. They were concerned about belongings falling and entering the robots’ machinery. Regarding system monitoring, the engineers were concerned about the intuitiveness and functionality of the control interface and whether it allowed operators to quickly identify faults or hazards, like battery fires or trip hazards, in a safe time limit to bring it to a stop. In terms of the retrieval area, collision risks—both robot‐robot and robot‐human—were another issue, as was travel speed and accessibility (some attendees may not have a smartphone with Bluetooth).

These questions are considered in light of existing literature, particularly the risks highlighted in current swarm logistics research (Milner et al., 2022; Abeywickrama et al., 2023, Carrillo‐Zapata et al., 2020; Jones et al., 2020, 2022). This case study responds to the call by Wardman and Mythen (2017) for “building up rich case study pictures” of the most effective strategies that might be used to deal with potentially harmful situations of future occurrences and developments where limited or partial information exists (Mythen et al., 2018: 97).

In the next section, the SOTEC framework is applied to the cloakroom case study and documents the findings of this process. Tables 1, 2, 3, 4, 5 capture the SOTEC framework's five sources of risk, outlining patterns of failure related to each risk source. Each risk source gave rise to multiple patterns of failure, but for the sake of space, only one illustrative pattern of failure per risk source is presented.

**TABLE 2:** Organizational sources of sociotechnical risk.

*Risks arising from interdependencies and interactions between different technical and social structures*		
Patterns of failure	Scenario example	Regulatory requirements
***Invisible Automation*** *Weaknesses or gaps in processes that maintain awareness, provide insight and issue alerts regarding the status, activities, and decisions of automated systems* (Macrae, 2022: 9)	A swarm robot has a significant error affecting its behavior, but this error is not being communicated effectively to its human operators For example, risks arise when a fault in the robot's obstacle avoidance system goes undetected, creating the potential for collisions	***Effective alerts*** The system shall have effective processes for continuously monitoring and transparently communicating system health to human operators
***Governance gaps*** *Gaps in organizational processes and systems that set standards for safety, monitor safety performance, and initiate action to address safety deficiencies* (Macrae, 2022: 9)	A swarm operator has insufficient training on how to respond to system malfunctions and assure the operational safety of the swarm system	***Access to governance and standards*** Operations shall require adequate training and guidance for swarm operators. For example, a safety plan or a standard for operating procedures that set out roles, responsibilities, and processes for the analysis and management of failures
***Regulatory voids*** *Absence of regulatory requirements, performance standards and associated oversight activities to assure the safe development, testing, deployment, and operation of automated systems*. (Macrae, 2022: 9)	A swarm system may be subject to little regulatory oversight with no regulatory system in place by the company/local councils for monitoring safety performance For example, a company deploying the swarm system may try to evade scrutiny from regulatory policies by employing self‐reporting practices (e.g., internal auditing), making it difficult for regulators to assess the performance of the swarm	***Regulative oversight mechanisms*** Ensure compliance with regulatory authorities and jurisdictions to prevent autonomous systems from evading oversight
***Supervisory degradation*** *Reduced or inadequate organizational arrangements to support, monitor, and assure the activities of supervising automated systems*. (Macrae, 2022: 9)	The organization has no processes for monitoring or promoting operator vigilance For example, risks arise when a lone system operator, after 9 h on duty, misses a failure indication	**Supervision** The system shall have in place rules and procedures to monitor and promote the vigilance of operators. Stipulating the duration of operator shifts, for instance, or mandating dual operators
**Competency limits** *Limitations or gaps in the roles, expertise, and experience available for analyzing and managing all aspects of safety across the development and deployment of an automated system*. (Macrae, 2022: 9)	The operating organization lacks personnel with responsibility for, or expertise commensurate with, managing the safety its swarm	***Safety expertise and Leadership*** The system operator shall have a safety team in place, with clear lines of responsibility and relevant competencies

**TABLE 3:** Technological sources of sociotechnical risk.

*Risks arising from the capabilities, affordances and constraints produced in and by material technologies*		
Patterns of failure	Scenario example	Regulatory requirements
**Automation immaturity**		**Automation maturity**
Automated systems regularly encounter situations, objects or hazards that are not recognized or are beyond the system's capabilities, leading to frequent failures (Macrae, 2022: 11)	Sensor systems may not reliably recognize or locate humans, especially those not carrying Bluetooth‐capable phones, leading to poor predictive behavior and increased collision risks	The system shall be effectively tested for its operational maturity and functionality. Limits shall be placed on its use that reflect its tested functionality (e.g., restrictions on people without a smart phone entering the system environment)
**Safety capability constraints**		**Unconstrained safety capability**
Technical features that reduce or constrain the safety capabilities of a system in order to optimize other aspects of system performance, efficiency, or experience (Macrae, 2022: 11)	A guidance algorithm designed to smooth the motion of agents unintentionally increases the swarm's reaction time to perceived hazards and hinders emergency stops	The system shall not constrain key safety capabilities. For example, by trading safety for optimized performance
**Hazard masking**		**Non‐masking of hazards**
Technical processes or features that result in hazards being inadvertently hidden, disguised, or rendered ambiguous (Macrae, 2022: 11)	The system's memory limitations hinder the retention and propagation of critical safety information	The system's design shall accommodate the identification, prioritization and retention of safety‐critical information
**Autonomy reliance**		**No self‐reliance on internal infrastructure**
Dependence on autonomous functionality for technical safety protections and controls, to the exclusion of other lower‐technology components that could provide safety redundancy” (Macrae, 2022: 11)	The swarm continues to function even if an individual robots’ “emergency obstacle avoidance” mechanism is disabled, relying only on the system‐level LiDAR for hazard detection and avoidance	The system shall become inert when the emergency avoidance system is disabled. Ideally it shall incorporate “passive” collision‐safety features requiring minimal activation (such as bumpers or zoning strategies)

**TABLE 4:** Epistemic sources of sociotechnical risk.

*Risks arising from the ways that knowledge and ignorance are constructed*		
Patterns of failure	Scenario example	Regulatory requirements
***Learning Lag***		**Time for learning**
*Operational and developmental activities exceed the capacity, systems, and resources available to analyze and learn from those activities and the surprises that they generate* (Macrae, 2022: 12)	The pace of system deployment creates data that exceeds the resources available to review and analyze it in a timely manner. As a result, significant “close‐calls” pass unreviewed for several days	The system shall be limited in its deployment to a pace that ensures that safety‐related data can be reviewed and responded to quickly
***Operational disengagement***		**Operational engagement**
*Limited or reduced efforts to gather data on, engage with, and make use of insights drawn from the operational experiences of people interacting with an autonomous system* (Macrae, 2022: 12)	The organization deploying the swarm fires several experienced swarm operators, diminishing their capacity to collect and analyze safety data	The system shall be overseen by experienced operators with sufficient capacity and expertise to identify and record significant behaviors
***Insensitivity to experience***		**Sensitivity to experience**
*Failure to anticipate, notice, or effectively explore the safety implications of events experienced during developmental, testing, or operational activities* (Macrae, 2022: 12)	Members of the public fail to queue in designed collection area, as anticipated. Swarm engineers and/or operators fail to adapt the system to accommodate this	The system shall be monitored in real‐world operation and revised to accommodate unanticipated behaviors
***Insufficient testing***		**Sufficient testing**
*Peripheral or limited use of different forms of simulation to explore, test, train, and improve the* behavior *of an autonomous system* (Macrae, 2022: 12)	Engineers overestimate maximum range of the LiDAR detection sensor by relying on a simulation that fails to fully represent real‐world noise	The system shall be subject to a rigorous and incremental testing regimen, which supplements computer simulations with lab‐tests and continues into the system's real‐world deployment phases
***Technical novelty***		**Transparent, cooperative, and open safety**
*Reluctance to create or share safety data due to fears it may disclose commercially sensitive information or reveal performance weaknesses in a competitive arena* (Macrae, 2022: 12)	The sharing of safety data is limited within the organization deploying the swarm, which manages reputational risks by restricting access to its safety incident database and discouraging full disclosure of safety‐related incidents	The system's operation shall be subject to requirements governing the reporting and sharing of safety data to ensure that limitations and gaps in safety performance are accurately documented and transparently circulated within the organization

**TABLE 5:** Cultural sources of sociotechnical risk.

*Risks arising from collective values, beliefs, norms, and practices*		
Patterns of failure	Scenario example	Regulatory requirements
***Performative production***		**Ongoing monitoring of, and critical reflection on, audit practices**
*Organizational attention and activities focus on maximizing performance on narrow public metrics which do not represent underlying quality, safety, or improvement of the system* (Macrae, 2022: 14)	The organization deploying the swarm focuses on increasing the number of containers collected, creating perverse incentives and corresponding behaviors, such as a shift to using one container per belonging instead of collecting multiple items in a single container	The system shall be assessed via audit practices that are themselves subject to critical review, aimed at identifying limitations and compensating for perverse consequences
***Existential pressure***		**Monitoring structural incentives**
*Overambitious targets and production pressures arising from fears that the existence of the organization is at stake in a competitive race to market* (Macrae, 2022: 14)	Market pressures incentivise the organization deploying the swarm to hurry deployment of the system, leading it to compromise on safety	The system shall be assessed in light of the structural incentives acting on its situational deployment
***Concern quashing***		**Encouraging operators to raise concerns and question decisions**
*Staff disempowered or discouraged in raising safety concerns, speaking up, or challenging assumptions due to fears of punitive responses or lack of support* (Macrae, 2022: 14)	Operators and/or managers are disincentivized from raising safety concerns. (e.g., by fear of not meeting targets, or of punitive action)	The system shall be managed via structures that encourage individuals to freely communicate safety concerns (e.g., nonpunitive channels for anonymous reporting)
***Developmental disintegration***		**Merging development activities**
*Norms and values that privilege rapid, widely distributed development and public‐domain testing and deprioritize integrated safety oversight and assurance* (Macrae, 2022: 14)	Swarm developers, primarily interested in engineering the system itself, move forward with deployment without developing sufficient testing and oversight practices	The system shall be subject to mandatory oversight milestones prior to deployment. Structures will be in place to develop oversight practices and coordinate their work with engineering development
***Presumptive reliability***		**Definitive reliability**
*Assumptions that human vigilance is effective for monitoring complex automated systems for long periods of time and requires little support or monitoring* (Macrae, 2022: 14)	Operators are expected to work alone, monitoring the system for long periods: a task that presupposes an unrealistic capacity for vigilance	The system shall not be predicated on unchallenged assumptions about the reliability and safety of human vigilance over prolonged periods of time. Systems and procedures will be implemented that aid operators to perform realistic oversight duties

## 4 FINDINGS

### 4.1 Structural sources of risk

Structural sources of risk arise from the interactions between human and nonhuman elements in a system (Table 1). For example, the consequences of a component‐level fault in an individual robot can be greatly amplified by the fault's system‐level effects. Consider a single faulty motor that immobilizes a robot in a location that blocks a pathway vital to the operation of the wider swarm. This blockage creates a “jam” that impedes robot‐robot and robot‐human traffic. This jam has the potential to escalate into a critical situation where other robots may give the faulty robot preference in the pathway, further cluttering up the environment in which the swarm is operating and hindering movement for both robots and human operators.

Regulation at this level is targeted at the identification and prevention of what Macrae (2022) calls “disruption amplifiers.” This may involve the development of system‐level tools, mechanisms, or processes capable of identifying robot‐level failures and preventing them from enlarging, expanding, or developing into wider disruptions.

Disruption amplifiers may also be thought of as a type of “interactive complexity,” where components may interact in unanticipated ways, perhaps because of failures or just because no designer anticipated the interactions that could occur (Perrow, 1999). For swarm engineers interested in mitigating this risk, a way of doing it might be to have some of the other robots in the swarm push the stationary robot to a safe location while communicating the failure to the wider swarm and signaling to its human operators (e.g., via hazard messages or activated warning lights).

### 4.2 Organizational sources of risk

Organizational sources of risk are those that arise from institutional structures, such as rules or expectations (Table 2). They might take the form of insufficient processes for communicating system information (status, activities, failures, decisions, etc.) to human operators, for example, or from insufficient processes for training human operators. For example, if a swarm robot experiences a major error while isolated from the group and lacks a system to alert human operators, it is improbable that the operators will be aware of the error. Although the swarm may be able to continue to perform efficiently without the robot, serious safety incidents could take place without going noticed (for instance, if a fault with the robot's obstacle avoidance system is not detected the robot has the potential to bump into people's shins). Macrae (2022: 2007) might call this type of organizational failure in a swarm context “invisible automation”—weaknesses or gaps in alerting processes that provide insight to operators regarding the status, activities, and decisions of automated system (Figure 2).

Due to the failure to provide an alert, there is a need for a specific regulation requiring the development of “effective alerts.” This involves designing organizational processes and systems that improve human performance and enhance interactions between humans and systems. This may take the form of rules directed at user interfaces and system ergonomics, for instance, or directed at the training, supervision, and workloads of human operators.

### 4.3 Technological sources of risk

Technological sources of risk arise from the specific capabilities and constraints of the engineered system (Table 3). For example, if the robots in a system are designed with insufficient memory to maintain adequate environmental awareness. This is especially the case when engineers produce individual robots that have limited memories so that when the robot detects or records a hazard (e.g., a dropped belonging), information about the hazard is deleted or overwritten by new information, and that critical information about the hazard gets lost. Considered in this way, building swarm robots with a limited memory (say, a robot that only retains information for 10 seconds before it gets overwritten or deleted) stops critical information about hazards or risks reaching the operators who are able to prevent accidents from happening. These are the kinds of technical hazards that are captured by conventional—non‐sociotechnical—risk analyses and are mostly included here for completion's sake.

Regulation at this level is targeted at ensuring the reliability and safe functioning of the system's internal architecture, usually via formal tools such as FEMA and PRA (Abeywickrama et al., 2023; Akhter et al., 2022; Farnell et al., 2019; Hernández‐Herrera et al., 2018). Macrae (2022) might call this type of technological risk in a swarm context “hazard masking” (Figure 2). As a result, hazard masking is tied to the production of a particular and specific regulatory requirement, namely, the project of engineering the non‐masking of hazards in the form of engineers building technical processes or features that do not inadvertently hide, disguise, or render ambiguous information. For instance, ensuring that robots are not built with limited memory and the system does not delete or overwrite information or that the system has some form of distributed method in place so that information can be effectively distributed across the swarm generating a “collective memory” (Wilson & Hauert, 2022: 6).

### 4.4 Epistemic sources of risk

Epistemic sources of risk arise from the ways that human knowledge is, by necessity, often incomplete, wrong, or out of date, creating uncertainties: pockets of ignorance that hide unexpected hazards (Table 4) (Downer, 2007, 2011, 2023; Sismondo, 1999). Some of these uncertainties involve “relevance” assumptions. For example, a system that is tested in a software simulation or in a controlled laboratory environment is vulnerable to uncertainties arising from the accuracy of the simulation and its ability to accurately reproduce the complexity of real‐world conditions. This might be the case where the engineer believes they have confidently calculated the maximum range of LiDAR scanning technology using the Gazebo 3D simulator but fails to take into consideration the “reality gap” of what this maximum range might look like in a real‐world hardware example.

For example, a 30 cm avoidance movement in the simulation may turn out to be 10 cm in reality because distance is something that is constructed between the engineer and the software, who tends to produce a simplified version of reality: in their creation, complexity and ambiguity are omitted in favor of turning the world into its computable double (Sismondo, 1999). Because of the engineer's focus on using one simulation software to explore, test, train, and improve the behavior of the swarm, they have inadvertently created an overreliance on one simulation software that lacks any form of rigorous testing. This overreliance on the simulation for testing means that the engineer is likely to believe that they have built a good avoidance system in simulation and testing when, in fact, the robot in the real world does not provide enough of a stopping distance to prevent a collision.

We call this type of epistemic risk in a swarm context “insufficient testing” and may also be thought as a type of “simulatory inattention” (Figure 2), however, our definition differs from Macrae (2022: 2010) in that testing must also be conducted outside simulation software and into the real world with hardware outside the laboratory environment.

Regulation at this level is targeted at testing and validation strategies, emphasizing caution regarding the fidelity of simulations and promoting incremental real‐world testing. As a result, insufficient testing is tied to the production of a particular and specific regulatory requirement, namely, the project of engineers conducting “sufficient testing,” in the form of producing knowledge about tests that is not partial, incorrect, or out of data and stopping pockets of ignorance to develop.

### 4.5 Cultural sources of risk

Cultural sources of risk arise from collective values, beliefs, norms, and practices that surround and inevitably shape the design and operation of autonomous systems (Table 5). For example, a culture of communication that discourages communication among engineers working on different aspects of a system's development may foster gaps in their understanding of the system's failure‐behavior. Such problems can originate on many levels and have many causes, from individual personalities to misaligned structural incentives (Reason, 1997). On a group level, for example, there could be an awareness of a faulty algorithm or safety problem that a group of swarm engineers wishes to resolve but may not communicate this to another team of swarm engineers external to their organization because they are in competition with their peers on the problem and see this as an opportunity to publish. Similarly, if a team of swarm operators has not yet developed a pipeline through which performance data is regularly updated and distributed across the development chain, then opportunities to improve oversight and assurance of the system will fall short because issues are unlikely to be resolved.

Macrae (2022: 2012) might call this type of cultural risk in a swarm context “developmental disintegration” (Figure 2)—where developers or operators who prioritize rapid development and deployment deprioritize integrated safety oversight and assurance. In order to avoid a culture of research communication that deemphasizes the importance of having an integrated, proactive, and centralized system for risk management, we argue that developmental disintegration can be tied to a specific regulatory requirement, namely, the project of “merging development activities,” in the form of developers and operators ensuring that there is proactive coordination between the multiple teams involved in different development and application activities. This inevitably involves work on multiple fronts, ranging from measures to shape or monitor organizational cultures (Downer, 2010) to stylized and mandated procedures for sharing and receiving certain types of information (Macrae, 2014). For instance, creating a culture that privileges the rapid and wide distribution of simulation and real‐world information and prioritizes consistent safety oversight and assurance pathways across various stakeholders (researchers, operators, and policymakers).

## 5 DISCUSSIONS AND LIMITATIONS

The research presented in this article is intended only to be illustrative: a way of exploring SOTEC's strengths and limitations and of trialing a potential means of implementation. The article explores the framework's value as a starting point for a theoretical conceptualization of autonomous swarm‐human risk's values and regulatory requirements.

As is hopefully apparent from the illustrative tables above, employing the SOTEC framework in the manner described promotes several important insights: First, it helps illustrate how social issues and the technology are interwoven to form a “sociotechnical system.” Second, it helps address risks that arise from the technology's sociotechnical nature: providing a systematic way of identifying patterns of failure that can emerge in its design, use, and control. Third, it helps identify and structure a broad range of regulatory targets (Winfield & Jirotka, 2017). Even if the SOTEC framework is a relatively blunt instrument; therefore, it offers a useful tool for stimulating awareness and discussion of nontechnical issues in safety assurance engineering.

In our specific case study of robot swarms in a public cloakroom, for example, it encouraged critical reflection on the constraints of their operational environment and on the needs and expectations of the people with whom they will interact. More generally, it highlighted a range of sociotechnical risks that could be useful in framing the swarm's regulatory requirements. By applying the SOTEC framework, with its five dimensions of sociotechnical risk, we were able to highlight several plausible patterns of failure that more conventional risk assessments might easily have overlooked. While not necessarily comprehensive, therefore, the framework was invaluable for developing a wide range of risk scenarios and corresponding regulatory targets in a more systematic manner than is conventional. Apart from two exceptions, the research team, in collaboration with the engineers and computer scientist recognized almost all the patterns of failure in the framework (22/24). Both of these exceptions—“simulatory inattention” and “competitive secrecy”—were recognized as epistemic sources of risk. However, simulatory inattention was changed to “insufficient testing” and competitive secrecy was changed to “technical novelty” in order to bring the swarm system in line with the swarm engineers’ grounded perception of the system being early on in the development process.

In conjunction with experts, we highlighted how these patterns of failure can shape the sociotechnical imagination and inform the development of risk scenarios in a systematic and rigorous manner. By means of the public cloakroom case study, we present 24 possible scenarios of such sociotechnical failures going forward, thus foregrounding SOTEC as a sociotechnical risk analysis method for autonomous systems such that it could be endorsed by a wide range of researchers situated in different domains (e.g., automotive) and real‐world applications (e.g., search and rescue). Additionally, we consider 24 possible regulatory requirements shaped by each pattern of failure and risk scenarios of the swarm operating in a public cloakroom.

Inevitably, there were meaningful limitations to our implementation strategy. One obvious line of critique, for instance, was its reliance on experts’ own assumptions about social and technical behavior. In helping frame and interpret our categories, the swarm engineers in our study drew on their own experiences, assumptions, and professional commitments, each with their own biases (Schwartz, 1996). (For instance, it is perhaps worth noting in this regard that all the engineers in our study were able‐bodied, with no evident cognitive or physical disabilities.) Further research is required to explore the significance of these kinds of limitations and means by which they might be mitigated when applying the framework.

## 6 CONCLUSION

Managing the risks of autonomous systems is increasingly becoming a priority of modern technology governance in a wide range of domains. This poses challenges, however, as such systems have unique affordances with complex nontechnical dimensions, and it is important that this is captured in their regulatory governance (Elish, 2019; Macrae, 2022; Winfield & Jirotka, 2017). As such, the regulation of autonomous systems risk arguably calls for a new phase in the evolution of sociotechnical risk research. The SOTEC framework offers a potential way by which research might begin to address this need. It creates a structured space wherein experts can imagine and explore the risks and requirements of autonomous systems in a way that attends to their nontechnical complexity. Developers of autonomous systems will also need to constantly scan the horizon for future AI techniques in the field of autonomous systems and consider future risks and opportunities in order to plan accordingly.

## CONFLICT OF INTEREST STATEMENT

The authors report no conflicts of interest in this work.

## Data Availability

The data that support the findings of this study are available on request from the corresponding author. The data are not publicly available due to privacy or ethical restrictions.

## Footnotes

1 The need for sociotechnical approaches to risk identification and analysis is well recognized in other technological contexts, as is evident in studies of safety‐critical systems in other domains (see, e.g., Perrow, 1999; Felt, 2016; Ionescu, 2020; Pence et al., 2020).

2 In this way, scenario analysis is similar to and connected to sociotechnical integration research (STIR), which uses a protocol (opportunity, considerations, alternatives, and outcome) to prompt researchers to think beyond the immediate technical aspects of a project and embed sociotechnical thinking within the day‐to‐day research and innovation lifecycle (Fisher, 2007; Fisher & Rip, 2013; Eden et al., 2013). Although SOTEC shares a common set of underlying principles to STIR, such as providing a structured approach to the identification and analysis of potential risks that can arise from the interaction between technological and social factors, SOTEC is better to use for our purpose because it is a theoretically informed and empirically grounded framework based on the failure of an autonomous system (i.e., the Uber AV crash) (Macrae, 2022).
